# The effect of vibrotactile feedback on postural sway during locomotor activities

**DOI:** 10.1186/1743-0003-10-93

**Published:** 2013-08-09

**Authors:** Kathleen H Sienko, M David Balkwill, Lars I E Oddsson, Conrad Wall

**Affiliations:** 1 Division of Health Sciences and Technology, Harvard University – Massachusetts Institute of Technology, Cambridge, MA, USA; 2 Jenks Vestibular Diagnostic Laboratory, Massachusetts Eye and Ear Infirmary, Boston, MA, USA; 3 Department of Mechanical Engineering, University of Michigan, Ann Arbor, MI, USA; 4 Department of Biomedical Engineering, University of Michigan, Ann Arbor, MI, USA; 5 Department of Otology & Laryngology, Harvard Medical School, Boston, MA, USA; 6 NeuroMuscular Research Center, Boston University, Boston, MA, USA; 7 Courage Kenny Research Center, Courage Kenny Rehabilitation Institute-part of Allina Health, Minneapolis, MN, USA

**Keywords:** Balance, Gait, Locomotion, Vibrotactile, Biofeedback, Vestibular, Sensory augmentation, Balance aid

## Abstract

**Background:**

Although significant progress has been achieved in developing sensory augmentation methods to improve standing balance, attempts to extend this research to locomotion have been quite limited in scope. The goal of this study was to characterize the effects of two real-time feedback displays on locomotor performance during four gait-based tasks ranging in difficulty.

**Methods:**

Seven subjects with vestibular deficits used a trunk-based vibrotactile feedback system that provided real-time feedback regarding their medial-lateral (M/L) trunk tilt when they exceeded a subject-specific predefined tilt threshold during slow and self-paced walking, walking along a narrow walkway, and walking on a foam surface. Two feedback display configurations were evaluated: the continuous display provided real-time continuous feedback of trunk tilt, and the gated display provided feedback for 200 ms during the period immediately following heel strike. The root-mean-square (RMS) trunk tilt and percentage of time below the tilt thresholds were calculated for all locomotor tasks.

**Results:**

Use of continuous feedback resulted in significant decreases in M/L trunk tilt and increases in percentage times below the tilt thresholds during narrow and foam trials. The gated display produced generally smaller changes.

**Conclusions:**

This preliminary study demonstrated that use of continuous vibrotactile feedback during challenging locomotor tasks allowed subjects with vestibular deficits to significantly decrease M/L RMS trunk tilt. Analysis of the results also showed that continuous feedback was superior.

## Findings

### Introduction

Sensory augmentation technologies were first developed as real-time balance aids for individuals with vestibulopathies. Proof of concept was demonstrated by using vibrotactile [1-6], electrotactile (lingual) [7,8], auditory [9-11], or visual [12] feedback displays. To date, however, few locomotor-based studies have been conducted. Dozza et al. [13] and Horak et al. [14] demonstrated reduced medial-lateral (M/L) trunk tilt during heel-to-toe walking with trunk-based vibrotactile biofeedback in subjects with unilateral vestibular loss. Hegeman et al. [15] reported that auditory biofeedback was not effective in reducing M/L sway during most gait tasks tested. The goal of this study was to compare the effects of two trunk-based vibrotactile feedback displays on M/L trunk tilt during a series of dynamic locomotor tasks performed by vestibulopathic subjects.

## Methods

Seven subjects (1 female, 6 males; 53.6 ± 12.9 yrs) with vestibulopathies diagnosed by a staff otoneurologist (Table 1) were recruited. All had used an earlier version of the vibrotactile feedback device [3-5]. Institutional Review Board approval conforming to the Helsinki Declaration was obtained and each subject gave informed consent prior to the study.

**Table 1:** Subject demographics

**Subject demographics**				**Computerized dynamic posturography**				**Rotation test**		**Classification**
**Subject ID**	**Age at clinical test**	**Age at research test**	**Gender**	**SOT score**	**SOT 5**	**SOT 6**	**MCT score**	**VOR Midrange gain**	**Time constant (s)**	**UVH or BVH**
1	56	61	M	73	35, 63, 70	56, 61, 45	148	0.786	7.76	UVH
2	56	61	M	70	52, 61, 69	17, 71, 46	155	0.856	6.54	UVH
3	31	32	M	46	Falls, Falls, Falls	Falls, Falls, Falls	151	0.514	0.44	BVH
4	50	66	F	49	Falls, Falls, Falls	Falls, Falls, Falls	161	0.86	N/A	UVH
5	60	61	M	70	Falls, 52, 64	66, Falls, 79	140	1.016	3.85	BVH
6	54	55	M	49	Falls, Falls, Falls	Falls, Falls, Falls	N/A	0.109	N/A†	BVH
7	37	39	M	46	Falls, Falls, Falls	Falls, Falls, Falls	138	0.625	0.27	BVH

Legend:

*SOT* Sensory Organization Test, Normal mean composite scores are 80 for 20-59 years old (yo) & 77 for 60-69 yo; 5th percentile (abnormal limits) scores are 69 for 20-59 yo & 70 for 60-69 yo.

*MCT* Motor Control Test, Normal mean composite scores are 143 for 20-59 yo & 152 for 60-69 yo; 5th percentile (abnormal limits) scores are 161 for 20-59 yo & 171 for 60-69 yo.

*N/A* Not available.

*VOR* Vestibuloocular reflex, as tested by 50 deg/sec peak sinusoidal vertical axis rotation, 0.01-1 Hz.

Midrange gain (0.2-1 Hz) and time constant estimated with parametric fit to gain and phase data (based on Dimitri et al., 1996).

† Response was too low for accurate estimation of time constant; classified as BVH by low VOR gain and low bilateral ice water calorics.

UVH or BVH, diagnostic classification as unilateral or bilateral vestibular hypofunction.

**Figure 1:**
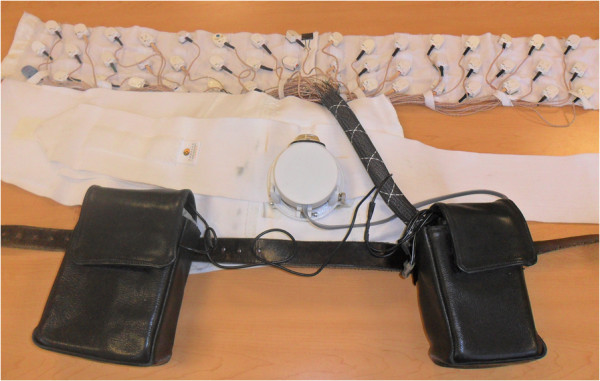
**Vibrotactile feedback device.** The vibrotactile display, elastic corset, IMU, PC104, and battery pack.

The vibrotactile feedback device (Figure 1) comprised a Honeywell HG1920 inertial measurement unit (IMU) mounted on the subject’s lower back, a central processing unit built on a PC104 platform communicating wirelessly with an external laptop, and a vibrotactile display. The IMU reported sway angles in the M/L and anterior-posterior (A/P) directions, and their vector sum (sway). The electromechanical actuators, referred to as tactors (Tactaid, Cambridge, MA), were activated at a frequency of 250 Hz. Columns of three tactors were mounted vertically on the left and right sides of the trunk to display M/L tilt in the corresponding directions. A tilt exceeding 1° (0.75° for one subject) activated the lowest tactor (low level), whereas a tilt exceeding 50% of the subject’s M/L limit of stability activated all three tactors (high level). Subjects were instructed to move in the direction opposite to the vibration to correct trunk tilt. In addition to the no vibrotactile feedback (tactors off) control condition, two vibrotactile feedback display configurations were evaluated: continuously displayed feedback (continuous), and feedback only for 200 ms beginning at the heel strike as detected by a predefined vertical acceleration threshold (gated). The gated feedback display, informed by Bent et al. [16], was designed to leverage the heel strike phase-dependent modulation of vestibular information during locomotion.

Prior to testing, subjects participated in a 45-minute training session. The tasks included self-paced walking (dimly lit visual target positioned at eye-level beyond end of walkway), slow-paced walking (fixed pace of 50-60 steps per minute maintained by a metronome), walking with narrowed base-of-support (20.5 – 30 cm wide), and walking across foam (7.3 m walkway of 10 cm thick medium density Sunmate Foam, Dynamic Systems, Inc., Leicester, NC). Subjects typically completed a set of three trials with the display off (baseline) followed by sets with continuous and gated feedback. A fourth set of trials was performed with tactors off for the narrowed and foam surfaces. The gated display was occasionally omitted from the foam surface protocol because heel strikes were not consistently detected when subjects gingerly placed their feet on the walkway.

Post-processing was performed using MATLAB (MathWorks, Natick, MA). The IMU raw data was low-pass filtered using a 4th order Butterworth filter (filtfilt.m). Gait initiation and termination were excluded prior to calculating all parameters. The root-mean-square (RMS) of M/L and A/P tilt and overall sway was calculated over a fixed distance for each trial. Percentage of time spent in each of the tactor activation zones (null, low, high) was also calculated on a trial-by-trial basis. Pacing information was approximated from the number of hand-counted gait cycles and the sampling rate. Individual repetitions comprising sets of similar display configurations were averaged on a subject-by-subject basis for each locomotion task. Parameter values were normalized for each subject as the percentage change from baseline (tactors off), and paired t-tests determined the effect of each display.

## Results

**Figure 2:**
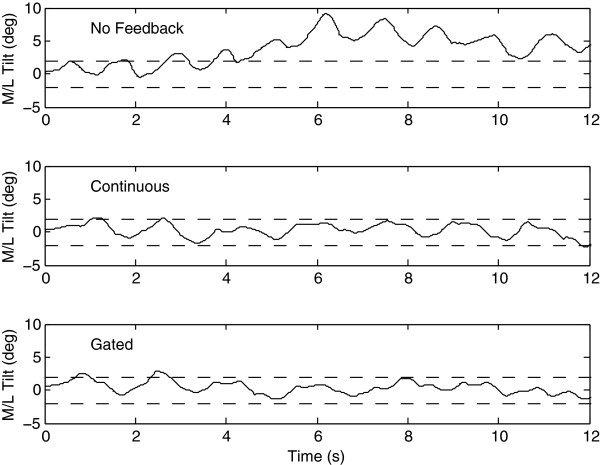
Sample data from subject 1 during self-paced walking showing M/L tilt with no feedback (top panel), continuous feedback (middle panel), and gated feedback (bottom panel).

Figure 2 shows the data for subject 1. Without vibrotactile feedback (top panel), this subject showed considerable lean toward the side of his lesion (right); with feedback (middle and bottom panels) he decreased his M/L tilt. In both feedback conditions, he was able to stay within the high threshold of ±2° (the threshold ranged from 1.5° - 3.5° across subjects).

No significant differences were observed between diagnostic groups, except for the percentage of time spent in the null and low zones combined during the walking with narrowed base-of-support task. UVH subjects had lower scores without feedback (p < .05) and greater improvements with feedback (p < .01) than their BVH counterparts. Due to the small number of subjects and the paucity of differences, the groups were pooled for the remaining analyses.

**Figure 3:**
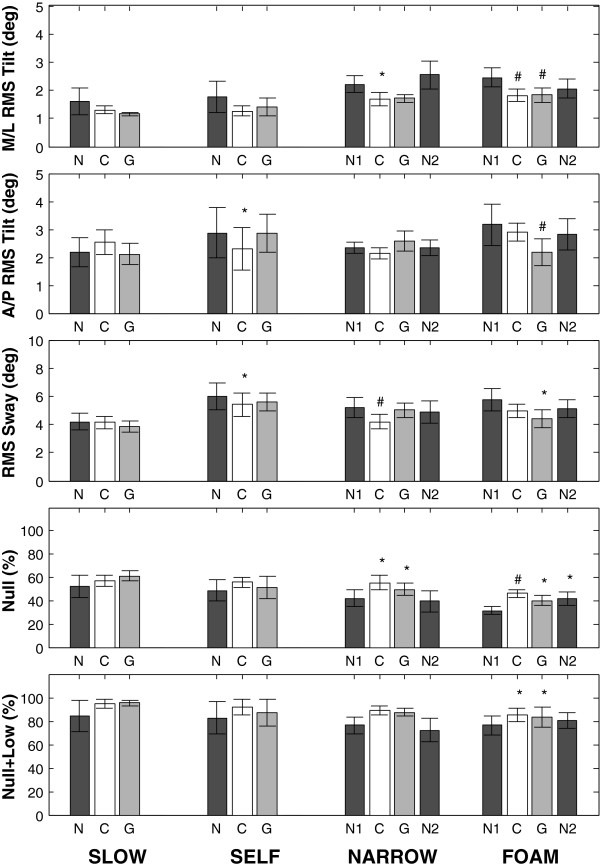
**Mean values for RMS roll, pitch, and tilt, as well as percentage of time with tilt less than the low threshold (Null) and less than the high threshold (Null+Low) for each of the four tasks (slow-paced and self-paced walking, walking on a narrow surface, and walking on foam).** Error bars indicate ±1 standard error of the mean. Displays with no feedback (N), continuous feedback (C), and gated feedback (G) were used for slow-paced and self-paced tasks. Narrow and foam surfaces were tested without feedback both before (N1) and after (N2) feedback trials. Statistical comparisons were made between the initial baseline values (N or N1) and the other displays (C, G, or N2). Significant effects are indicated for p < .05 (*) and p < .01 (#).

Figure 3 shows that, regardless of display type, M/L RMS tilt decreased for all locomotor tasks when feedback was provided. Improvements were more pronounced as the task difficulty increased, and were statistically significant for narrow (p < .05) and foam (p < .01) surfaces. RMS sway demonstrated a similar pattern, whereas the effects on A/P tilt were inconsistent. Percentage of time spent in the null zone as well as in the null and low zones combined increased with feedback, and significantly improved for the narrow and foam tasks (p < .05). Feedback benefits were apparent for all subjects, with each reducing M/L RMS tilt by more than 25%, and increasing null zone time by more than 15% during at least one of the difficult tasks (narrow and foam). For each task, pace changed by less than 2% with feedback (group average), which was insignificant. Overall, pace increased above baseline with feedback for 50% of subjects and 41% of trials. In general, continuous feedback showed greater improvements than gated feedback, but these differences were not statistically significant. Performance improved overall for the second set of trials without feedback in comparison with the first set; however, this difference was not statistically significant.

## Discussion

Vibrotactile feedback improved subjects’ control of M/L tilt without sacrificing pace, which suggested that subjects had adequate time to perceive, process, and respond to the vibrotactile cues given the training received. Improved control was more apparent during the more difficult tasks (narrow and foam), most likely due to the greater room for improvement on the baseline metrics. Since these two “difficult” tasks were performed near the end of the session, some of the improvement was attributed to subjects’ increased experience.

The continuous display configuration has the advantage of delivering information during both single and double support phases, which allows postural corrections to be implemented throughout the complete gait cycle. The gated feedback display configuration provided information about M/L tilt such that an M/L foot placement correction could be made on the subsequent step. Bent et al. [16], who delivered galvanic vestibular stimulation (GVS) during heel strike, mid-stance, and toe-off, found the largest changes in foot placement when GVS was delivered at heel strike. It should be noted that shorter delays are associated with GVS than with receiving, processing, and acting on vibrotactile tilt information provided to the trunk. Additionally, the response to GVS is involuntary, whereas responding to vibrotactile feedback is assumed to be mainly voluntary.

Subjects expressed a slight preference for continuous feedback across all locomotor tasks. They verbally indicated an increased level of confidence when tilt information was continuously displayed. In fact, they questioned the device’s status during the gated display trials when no vibrotactile cue was received when walking more than a few steps. Subjects’ preference for the gated feedback was based on the notion that their attention could be focused on the device only when a balance crisis occurred. Subjects preferring the continuous display stated that they felt more comfortable because they were receiving the most complete information.

One drawback of the gated display was the simple threshold-based algorithm for detecting the elevated vertical accelerations of the trunk at the heel strike events. This algorithm worked reliably during self-paced trials. However, when subjects employed a slower gait during slow-paced walking and narrow stance walking, the vertical accelerations decreased and the threshold was less sensitive. The worst case occurred during foam walking because the foam dampened the vertical accelerations. Vertical accelerations were also dependent on the amount of cushioning in a subject’s footware. Study limitations included a small sample size and a short training session. Given the small sample size, the UVH and BVH results were combined; in practice, clinicians may treat these patients differently with respect to compensation, adaptation, and substitution strategies [17].

During the training sessions, stiffening in the coronal plane, i.e., rigid and awkward gait, was observed, especially among subjects who were intent on preventing the device from vibrating. Extra time and coaching had to be provided to ensure that they used the vibrotactile feedback to augment their natural gait. This condition was a primary reason for using the lowest row of tactors to indicate a slight tilt, and the stronger stimulus (all three rows activated simultaneously) to indicate a more severe tilt. Subjects were told that they could expect, and should feel, the lower level activating twice per gait cycle.

## Conclusion

This preliminary study showed that individuals with vestibular deficits can decrease M/L RMS trunk tilt during challenging locomotor tasks with the use of real-time vibrotactile feedback.

## Abbreviations

A/P: Anterior-posterior; BVH: Bilateral vestibular hypofunction; GVS: Galvanic vestibular stimulation; IMU: Inertial measurement unit; M/L: Medial-lateral; RMS: Root-mean-square; UVH: Unilateral vestibular hypofunction.

## Competing interests

The authors state that C. Wall is an inventor on an issued patent and has equity interest in BalanceTek, Inc.

## Authors’ contributions

KHS designed the study, carried out the study, analyzed the data, interpreted the data, and drafted the manuscript. MDB developed the experimental instrumentation and software, designed the study, analyzed the data, interpreted the data, and drafted the manuscript. LIEO participated in the study and instrumentation design and helped to draft the manuscript. CW conceived of the study, participated in its design and coordination, and helped to draft the manuscript. All authors read and approved the final manuscript.
